# Up-and-down immunity of pregnancy in humans

**DOI:** 10.12688/f1000research.11690.1

**Published:** 2017-07-25

**Authors:** Philippe Le Bouteiller, Armand Bensussan

**Affiliations:** 1 INSERM U976, Université Paris Diderot, Sorbonne Paris Cité, Hôpital Saint-Louis, Equerre Bazin, 1, Avenue Claude Vellefaux, 75475 Paris Cedex 10, France

**Keywords:** decidua basalis, maternal immune system, immune tolerance

## Abstract

One part of the human placenta in early pregnancy is particularly important for local immunity: the *decidua basalis*, which is transformed endometrium located at the site of embryo implantation *. *This placental bed tissue contains both maternal uterine immune cells, including decidual natural killer (NK) cells, the dominant leukocyte population exhibiting a unique phenotype, and fetal extravillous trophoblast which comes into direct contact with maternal decidual cells *.* To establish a successful placental development and healthy pregnancy outcome, the maternal immune system must tolerate paternal antigens expressed by trophoblast cells yet remain efficient for clearing any local pathogen infection. This review deals mainly with decidual NK cells. A key element, among others, to achieve such dual functions is the direct interaction between activating and inhibitory receptors expressed by decidual NK cells and their specific ligands presented by trophoblast or other decidual cells. Depending whether maternal decidual cells and trophoblast are infected by viruses, the balance between activating and inhibitory receptor signals mediated by decidual NK cell–trophoblast cross-talk results in tolerance (healthy pregnancy) or specific killing (pathogen-infected cells).

## Introduction

The human placenta consists of two components—a fetal and a maternal one—that must interact successfully for a healthy pregnancy outcome ^1^. The maternal component is the endometrium, which undergoes modifications to form the *decidua basalis* from the very earliest stages of pregnancy. The only fetal component is the trophoblast in contact with maternal immune cells, constituting the junction between two immunologically distinct individuals (mother and fetus). A recent report has developed a thorough classification system based on specific markers to identify the different trophoblast subpopulations in the first trimester of pregnancy ^2^. Invasive extravillous cytotrophoblast (EVCT) is present in the *decidua basalis* ^3, 4^. Differing from the *decidua basalis*, the *decidua parietalis* lines the uterine cavity and is in direct contact with HLA-G ^+^ trophoblast in the chorion laeve with no invasion in spiral arteries ^4^. EVCT is also present in the distal ends of spiral arteries in contact with maternal blood, contributing to the uterine vascular remodeling ^5– 7^. Such remodeling is critical to ensure optimal utero-placental blood flow during pregnancy. In early pregnancy decidua, EVCT comes into close contact with various maternal immune cells: prominent granulated decidual natural killer (dNK) cells, macrophages, CD4 ^+^ T cells, including T helper 17 (Th17) cells ^8^, and CD8 ^+^ T cells ^4^. Another maternal-fetal interface is constituted by the multinucleated syncytiotrophoblast, the outer part of chorionic villi, which is in contact with maternal immune cells present in the maternal blood of the intervillous blood space ^9^. This review focuses mostly on the role of EVCT and dNK cells in pregnancy immunity.

During pregnancy, the maternal immune system responds to fetal trophoblast antigens. In particular, it produces maternal anti-paternal antibodies, but oddly these antibodies are not harmful for the fetus ^10– 12^. Furthermore, in normal pregnancy, trophoblast is never destroyed by maternal decidual immune cells ^13^, suggesting the occurrence of very efficient immunosuppressive mechanisms. Yet recent findings demonstrate that viral infection awakes maternal decidual immunity to rapidly clear pathogens and avoid further spreading to the fetus ^6, 14, 15^. These intriguing, contradictory observations raise the following question: how do the interactions between maternal immune cells and fetal trophoblast either lead to downregulation of maternal anti-paternal immunity or trigger efficient maternal immunity against decidual pathogen-infected cells? The aim of this review is to analyze some of the recent scientific advances that contribute to explain these intriguing, contradictory observations. One key element of such up-and-down local decidual immunity lies in the specific receptor-ligand interactions occurring at the cell surface of maternal decidual immune cells and fetal trophoblast. Among the different control mechanisms described to date, the crucial role exerted by dNK cells in both healthy and virus-infected pregnancies will be the focus of this review.

## Healthy pregnancy: down-modulated adaptive and innate decidual immunity

### Unique array of HLA class I molecules on trophoblast

In contrast to most somatic cells, infiltrating EVCT cells do not express the polymorphic T-cell ligands HLA-A and HLA-B class I ^16– 18^ nor do they express HLA class II molecules ^17^. The absence of polymorphic HLA-A and HLA-B expression thus limits fetal antigen presentation by non-infected EVCT to the maternal CD8 ^+^ cytotoxic T cells ^19, 20^. Moreover, it has been shown that cytotoxic T cells in the decidua express less perforin and granzyme B than CD8 ^+^ T cells present in the peripheral blood (PB) ^21, 22^. This is one of the various mechanisms used by the fetus for avoiding killing by CD8 ^+^ T cells of the invading EVCT which plays a crucial role in uterine vascular remodeling and placental development ^5^. EVCT cells express the fetal polymorphic class I molecule, HLA-C, only in the β2-microglobulin-associated form and at a high level ^22– 24^. HLA-C is the ligand of activating or inhibitory killer-cell immunoglobulin receptors (KIRs). Engagement of these inhibitory receptors by specific, and so-far-unknown, trophoblast ligands downregulates the cytotoxic function of dNK cells ^15^. In addition, EVCT expresses the non-polymorphic HLA-E, HLA-G, and HLA-F ^7, 25^. HLA-E is the ligand of CD94/NKG2A heterodimer inhibitory dNK cell receptor (see next section). HLA-G molecules are expressed at the cell surface of EVCT with a prolonged half-life ^6^. HLA-G is the ligand of LILRB1 inhibitory receptor present on about 40% of dNK cells ^18^. LILRB1 has a high affinity for the dimeric form of HLA-G on EVCT ^18^. Such strong binding contributes to limit the cytotoxic function of dNK cells ^26^. This is an additional inhibitory mechanism. Syncytiotrophoblast, the outer part of chorionic villi, in contact with the maternal blood of the intervillous space, is devoid of classical and non-classical HLA class I surface expression ^17^. Thus, it cannot present fetal antigens to the maternal CD8 ^+^ T cells present in the intervillous space. Though a matter of debate ^27, 28^, some reports indicate that syncytiotrophoblast produces soluble HLA-G ^29, 30^. Soluble HLA-G might exert an immunosuppressive protective role in pregnancy ^19^ by triggering the killing of maternal activated CD8 ^+^ T cells present in the *decidua basalis* as well as in the maternal blood of the intervillous space ^31^.

### Decidual natural killer cells in healthy pregnancy: lack of cytotoxic function

dNK cells constitute the great majority of maternal immune cells present in early *decidua basalis* (about 70% of leukocytes present), where the EVCT infiltrates into maternal tissue. The dNK phenotype in healthy pregnancy is unique, differing from PB NK cells ^14^. The major dNK subpopulation is characterized as CD56 ^bright^/CD16 ^−^/CD160 ^−^ (non-cytotoxic phenotype), whereas about 90% of the PB NK cells are CD56 ^dim^/CD16 ^+^/CD160 ^+^ (cytotoxic phenotype) ^32^. Although their abundant intracellular lytic granules contain perforin, granzyme, and granulysin ^33^, dNK cells are poorly cytotoxic ^9^. In contrast to circulating NK cells, dNK cells display a poor ability to kill various non-infected cell target lines, including human K562 and .221 as well as murine P815 cell lines ^34^. Other dNK cell functions—regulation of trophoblast invasion and subsequent spiral artery remodeling—are controlled by dNK cell receptors that interact with specific HLA class I or non-major histocompatibility complex (non-MHC) class I ligands present on EVCT ^35^. Similarly, in mice, MHC molecules were shown to educate uterine NK cells to trigger uterine arterial remodeling ^36^. Although dNK cells express several activating receptors, including NKp46, NKp30, NKG2D, and CD94/NKG2C, and some activating receptors of the KIR family, they are not cytotoxic against EVCT ^13^. Several studies have characterized the different mechanisms preventing the dNK cells from exerting cytotoxic function. First, this is due to specific interactions between dNK inhibitory receptors and specific ligands. We reported that the engagement of CD94/NKG2A inhibitory receptor with its specific ligand HLA-E expressed by EVCT is a dominant negative regulatory mechanism that prevents cytotoxicity toward trophoblast ^37^. Second, another inhibitory mechanism is provided by engagement of the LILRB1 inhibitory receptor by its specific HLA-G ligand expressed at the cell surface of EVCT ^38^. Third, a report demonstrated that VEGF-C secreted by dNK cells in the first trimester of pregnancy triggered upregulation of TAP-1 in EVCT, protecting them from a cytotoxic role ^39^. Thus, in normal pregnancy, there is an obvious resistance to dNK cell killing function, although dNK cells are potentially capable of cytolytic activity ^37^.

## Pathogen-infected pregnancy: awakened decidual immunity

### Contact with autologous decidual human cytomegalovirus-infected cells restores decidual natural killer cell killing potential

Human pregnancy is characterized by tolerance to the fetus through down-local decidual immunity but balanced with fetal decidual defense against pathogens. dNK cells are poorly cytotoxic in healthy pregnancy yet possess a likely functional lytic machinery ^14^. Indeed, using a redirected cell lysis assay, a study reported that specific engagement of NKp46 and, to a lesser extent, NKp30 activating receptors on isolated dNK cells from early decidua induced P815 cell target lysis ^37^. Human cytomegalovirus (HCMV) infection modulates the dNK cell receptor repertoire. Co-culture of dNK cells with HCMV-infected autologous stromal cells increased the number of CD56 ^dim^ dNK cells associated with the appearance of CD16 and NKG2C activating receptors ^40^. Such a phenotype is consistent with the acquisition of a cytotoxic profile. Similarly, isolated dNK cells infected with *Toxoplasma gondii* were shown to express markers of cytotoxicity, including increased expression of CD16 and NKG2D receptors, and to acquire a CD56 ^dim^ phenotype ^41^. These different observations indicated that dNK cells acquire cytotoxic potential in the pregnant uterus when the decidua is infected by pathogens. dNK cells become cytotoxic when in contact with HCMV-infected autologous decidual stromal cells ^42^. dNK cells engage immune synapse with HCMV-infected autologous stromal cells and polarize their lytic machinery toward infected cells. In contrast, purified dNK cells in contact with uninfected autologous stromal cells do not exert their killing function ^40^. Furthermore, via immunohistochemistry, the presence of dNK cells was observed in the vicinity of HCMV-positive cells in placental tissue obtained after elective pregnancy termination ^40^. Another article indicated that isolated dNK cells expressing KIR2DS1 activating receptor acquired higher cytotoxic function than KIR2DS1 ^negative^ dNK cells when in contact with HCMV-infected decidual stromal cells ^13^. Moreover, a recent study reported that a structural modulation of HLA-C is required for a potent KIR2DS1-mediated NK cell activation ^43^. Whether such HLA-C modulation could occur at the cell surface of HCMV-infected EVCT in pregnancy remains to be determined. Thus, dNK cells were shown to clearly exert a cytotoxic function in the pregnant uterus when the decidua is infected by pathogens.

A controversial question can legitimately arise from the above results obtained from *in vitro* co-culture of isolated dNK cells and stromal cells from the same early pregnancy sample: is there a bias due to the limited type of decidual cells surrounding dNK cells in these *in vitro* experiments? The beginning of an answer has been provided by a recent article ^44^. The authors used a multi-cell-type solid placental tissue containing a variety of immune and non-immune cells, including fetal and maternal cells which more closely resemble infection *in vivo*. In the latter study, it was demonstrated that HCMV infection triggered a robust interferon-gamma (IFN-γ) and IFN-γ-inducible protein-10 (IP-10) release by dNK cells, thus differing from another study that used *in vitro* co-culture with only two cell components: autologous dNK and decidual stromal cells ^40^. In the latter case, no upregulation of IFN-γ but rather down-modulation of IP-10 by dNK cells was observed ^40^. These different results demonstrate the crucial influence of the cellular microenvironment.

Decidual CD8 ^+^ T cells present in early pregnancy are fully functional ^15^. They can recognize fetal HLA-C expressed at high levels on the cell surface of EVCT ^6, 20^. HLA-C expressed by EVCT thus exerts a dual role: it can present pathogen-derived peptides to decidual CD8 ^+^ T cells when EVCT is infected. HLA-C expressed by EVCT is also a specific ligand for various activating KIR dNK receptors ^24^. Such interactions result in the stimulation of dNK cell cytotoxic function ^15^. Whether virus-specific and HLA-C restricted decidual CD8 ^+^ T cells are present in infected decidua and able to kill infected cells remains to be determined. Detection of virus-specific CD8 ^+^ T cells in decidual tissue ^45^ suggests that this might be the case. Moreover, a recent study demonstrated that activation of dNK cells through KIR2DS4 activating receptor (which also binds HLA-C) stimulates EVCT migration needed for subsequent placental development ^46^.

## Concluding remarks

During pregnancy, there is a conflicting need for both reproductive success and protection against congenital transmission of viruses and other pathogens. To date, many mechanisms have been described that contribute to block potentially harmful maternal–anti-paternal immune response ^19^. Until recently, little was known about the ways used by the maternal immune system to eradicate local decidual infection. dNK cells present in large numbers in *decidua basalis* play different roles at the beginning of pregnancy by interacting with specific EVCT-bound ligands, including HLA class I. On the one hand, dNK cells are poorly cytotoxic, thus preventing the killing of EVCT. On the other hand, dNK cells acquire cytotoxic potential when they interact with infected decidual cells. Besides, the immunity of pregnancy requires the involvement of other limbs of the immune system ^47^. Indeed, several studies have identified additional mechanisms that contribute to T-cell tolerance in the *decidua basalis* ^6, 48^. Several other innate and adaptive immune interactions occur at the maternal-fetal interface ^49^. Important to consider is the role of regulatory T cells ^50– 52^ as well as Th17 cells in the human decidua ^8^. Whether the same dNK-mediated target recognition similarly blocks other viral congenital infections that may occur is yet to be elucidated. It would be important to study how local immunity in pregnancy may control infections of hepatitis C virus ^53^, human immunodeficiency virus ^54^, Zika virus ^55^, and *T. gondii* parasite ^41^.

## References

[1] Burton GJ Jauniaux E: What is the placenta? *Am J Obstet Gynecol.* 2015;213(4 Suppl):S6.e1, S6–8. 10.1016/j.ajog.2015.07.050 26428504

[2] Lee CQ Gardner L Turco M: What Is Trophoblast? A Combination of Criteria Define Human First-Trimester Trophoblast. *Stem Cell Reports.* 2016;6(2):257–72. 10.1016/j.stemcr.2016.01.006 26862703 PMC4750161

[3] Le Bouteiller P Blaschitz A: The functionality of HLA-G is emerging. *Immunol Rev.* 1999;167(1):233–44. 10.1111/j.1600-065X.1999.tb01396.x 10319265

[4] Bulmer JN Williams PJ Lash GE: Immune cells in the placental bed. *Int J Dev Biol.* 2010;54(2–3):281–94. 10.1387/ijdb.082763jb 19876837

[5] Lippe EM Yamada AT Sharma S: Human uterine Natural Killer cells: friends or foes of pregnancy outcomes. In *Immunology of pregnancy.*Gerard Chaouat, Nathalie Lédée, editor Bentham Science Publishers,2013;360–376. 10.2174/9781608057337113010018

[6] Moffett A Colucci F: Co-evolution of NK receptors and HLA ligands in humans is driven by reproduction. *Immunol Rev.* 2015;267(1):283–97. 10.1111/imr.12323 26284484

[7] Faas MM de Vos P: Uterine NK cells and macrophages in pregnancy. *Placenta.* 2017;56:44–52, pii: S0143-4004(17)30179-0. 10.1016/j.placenta.2017.03.001 28284455

[8] Piccinni MP Lombardelli L Logiodice F: T helper cell mediated-tolerance towards fetal allograft in successful pregnancy. *Clin Mol Allergy.* 2015;13(1):9. 10.1186/s12948-015-0015-y 26064081 PMC4461901

[9] Gaynor LM Colucci F: Uterine Natural Killer Cells: Functional Distinctions and Influence on Pregnancy in Humans and Mice. *Front Immunol.* 2017;8:467. 10.3389/fimmu.2017.00467 28484462 PMC5402472

[10] Margni RA Binaghi RA: Nonprecipitating asymmetric antibodies. *Annu Rev Immunol.* 1988;6:535–54. 10.1146/annurev.iy.06.040188.002535 3289577

[11] Moffett A Loke C: Immunology of placentation in eutherian mammals. *Nat Rev Immunol.* 2006;6(8):584–94. 10.1038/nri1897 16868549

[12] Girardi G Bulla R Salmon JE: The complement system in the pathophysiology of pregnancy. *Mol Immunol.* 2006;43(1–2):68–77. 10.1016/j.molimm.2005.06.017 16023727

[13] Crespo ÂC, Strominger JL Tilburgs T: Expression of KIR2DS1 by decidual natural killer cells increases their ability to control placental HCMV infection. *Proc Natl Acad Sci U S A.* 2016;113(52):15072–7. 10.1073/pnas.1617927114 27956621 PMC5206558

[14] Le Bouteiller P: Human decidual NK cells: unique and tightly regulated effector functions in healthy and pathogen-infected pregnancies. *Front Immunol.* 2013;4:404. 10.3389/fimmu.2013.00404 24324468 PMC3839044

[15] Crespo ÂC, van der Zwan A Ramalho-Santos J: Cytotoxic potential of decidual NK cells and CD8+ T cells awakened by infections. *J Reprod Immunol.* 2017;119:85–90. 10.1016/j.jri.2016.08.001 27523927 PMC5290261

[16] Apps R Murphy SP Fernando R: Human leucocyte antigen (HLA) expression of primary trophoblast cells and placental cell lines, determined using single antigen beads to characterize allotype specificities of anti-HLA antibodies. *Immunology.* 2009;127(1):26–39. 10.1111/j.1365-2567.2008.03019.x 19368562 PMC2678179

[17] Le Bouteiller P: Major Histocompatibility Complex Class I unique expression in human trophoblasts: facts, questions and controversies. In *Immunology of pregnancy 2013* Gérard Chaouat, Olivier Sandra, Nathalie Lédée, editor Bentham Science Publishers,2013b;158–173. Reference Source

[18] Sharkey AM Xiong S Kennedy PR: Tissue-Specific Education of Decidual NK Cells. *J Immunol.* 2015;195(7):3026–32. 10.4049/jimmunol.1501229 26320253 PMC4574523

[19] Chaouat G: "Tolerance" to the fetal allograft?In *Immunology of pregnancy 2013* G. Chaouat, O. Sandra, and N. Lédée, editors. Bentham Science Publishers,2013;466–526. Reference Source

[20] Tilburgs T Strominger JL: CD8+ effector T cells at the fetal-maternal interface, balancing fetal tolerance and antiviral immunity. *Am J Reprod Immunol.* 2013;69(4):395–407. 10.1111/aji.12094 23432707 PMC3711858

[21] Tilburgs T Schonkeren D Eikmans M: Human decidual tissue contains differentiated CD8 ^+^ effector-memory T cells with unique properties. *J Immunol.* 2010;185(7):4470–7. 10.4049/jimmunol.0903597 20817873

[22] Moffett A Colucci F: Uterine NK cells: active regulators at the maternal-fetal interface. *J Clin Invest.* 2014;124(5):1872–9. 10.1172/JCI68107 24789879 PMC4001528

[23] Moffett A Loke C McLaren A: Biology and pathology of trophoblast. Cambridge University Press, Cambridge, UK.2006;272 10.1017/CBO9780511545207

[24] Chazara O Xiong S Moffett A: Maternal KIR and fetal HLA-C: a fine balance. *J Leukoc Biol.* 2011;90(4):703–16. 10.1189/jlb.0511227 21873457

[25] Hackmon R Pinnaduwage L Zhang J: Definitive class I human leukocyte antigen expression in gestational placentation: HLA-F, HLA-E, HLA-C, and HLA-G in extravillous trophoblast invasion on placentation, pregnancy, and parturition. *Am J Reprod Immunol.* 2017;77(6):e12643. 10.1111/aji.12643 28185362

[26] Carosella ED LeMaoult J: HLA-G: a look back, a look forward. *Cell Mol Life Sci.* 2011;68(3):337–40. 10.1007/s00018-010-0577-2 21088981 PMC11114643

[27] Sargent IL: Does 'soluble' HLA-G really exist? Another twist to the tale. *Mol Hum Reprod.* 2005;11(10):695–8. 10.1093/molehr/gah196 16330473

[28] Ferreira LM Meissner TB Tilburgs T: HLA-G: At the Interface of Maternal-Fetal Tolerance. *Trends Immunol.* 2017;38(4):272–86. 10.1016/j.it.2017.01.009 28279591

[29] Le Bouteiller P: Human villous trophoblast and the lack of intron 4-retaining soluble HLA-G secretion: beware of possible methodological biases. *Mol Hum Reprod.* 2005;11(10):711–3. 10.1093/molehr/gah211 16330471

[30] Hunt JS Geraghty DE: Soluble HLA-G isoforms: technical deficiencies lead to misinterpretations. *Mol Hum Reprod.* 2005;11:715–7. 10.1093/molehr/gah223 16330470

[31] Solier C Aguerre-Girr M Lenfant F: Secretion of pro-apoptotic intron 4-retaining soluble HLA-G1 by human villous trophoblast. *Eur J Immunol.* 2002;32(12):3576–86. 10.1002/1521-4141(200212)32:12<3576::AID-IMMU3576>3.0.CO;2-M 12516543

[32] Le Bouteiller P Tabiasco J Polgar B: CD160: a unique activating NK cell receptor. *Immunol Lett.* 2011;138(2):93–6. 10.1016/j.imlet.2011.02.003 21324341

[33] Veljkovic Vujaklija D Dominovic M Gulic T: Granulysin expression and the interplay of granulysin and perforin at the maternal-fetal interface. *J Reprod Immunol.* 2013;97(2):186–96. 10.1016/j.jri.2012.11.003 23399514

[34] Kopcow HD Allan DS Chen X: Human decidual NK cells form immature activating synapses and are not cytotoxic. *Proc Natl Acad Sci U S A.* 2005;102(43):15563–8. 10.1073/pnas.0507835102 16230631 PMC1266146

[35] Hanna J Goldman-Wohl D Hamani Y: Decidual NK cells regulate key developmental processes at the human fetal-maternal interface. *Nat Med.* 2006;12(9):1065–74. 10.1038/nm1452 16892062

[36] Kieckbusch J Gaynor LM Moffett A: MHC-dependent inhibition of uterine NK cells impedes fetal growth and decidual vascular remodelling. *Nat Commun.* 2014;5: 3359. 10.1038/ncomms4359 24577131 PMC3948146

[37] El Costa H Casemayou A Aguerre-Girr M: Critical and differential roles of NKp46- and NKp30-activating receptors expressed by uterine NK cells in early pregnancy. *J Immunol.* 2008;181(5):3009–17. 10.4049/jimmunol.181.5.3009 18713971

[38] Favier B Lemaoult J Lesport E: ILT2/HLA-G interaction impairs NK-cell functions through the inhibition of the late but not the early events of the NK-cell activating synapse. *FASEB J.* 2010;24(3):689–99. 10.1096/fj.09-135194 19841038

[39] Kalkunte SS Mselle TF Norris WE: Vascular endothelial growth factor C facilitates immune tolerance and endovascular activity of human uterine NK cells at the maternal-fetal interface. *J Immunol.* 2009;182(7):4085–92. 10.4049/jimmunol.0803769 19299706 PMC3616376

[40] Siewiera J El Costa H Tabiasco J: Human cytomegalovirus infection elicits new decidual natural killer cell effector functions. *PLoS Pathog.* 2013;9(4):e1003257. 10.1371/journal.ppat.1003257 23592985 PMC3617138

[41] Xu X Fu Q Zhang Q: Changes of human decidual natural killer cells cocultured with YFP- *Toxoplasma gondii*: implications for abnormal pregnancy. *Fertil Steril.* 2013;99(2):427–32. 10.1016/j.fertnstert.2012.09.016 23089237

[42] Le Bouteiller P Siewiera J Casart Y: The human decidual NK-cell response to virus infection: what can we learn from circulating NK lymphocytes? *J Reprod Immunol.* 2011;88(2):170–5. 10.1016/j.jri.2010.12.005 21277025

[43] van der Ploeg K Chang C Ivarsson MA: Modulation of Human Leukocyte Antigen-C by Human Cytomegalovirus Stimulates KIR2DS1 Recognition by Natural Killer Cells. *Front Immunol.* 2017;8:298. 10.3389/fimmu.2017.00298 28424684 PMC5372792

[44] Weisblum Y Panet A Zakay-Rones Z: Human cytomegalovirus induces a distinct innate immune response in the maternal-fetal interface. *Virology.* 2015;485:289–96. 10.1016/j.virol.2015.06.023 26318261

[45] van Egmond A van der Keur C Swings GM: The possible role of virus-specific CD8 ^+^ memory T cells in decidual tissue. *J Reprod Immunol.* 2016;113:1–8. 10.1016/j.jri.2015.09.073 26496155

[46] Kennedy PR Chazara O Gardner L: Activating KIR2DS4 Is Expressed by Uterine NK Cells and Contributes to Successful Pregnancy. *J Immunol.* 2016;197(11):4292–300. 10.4049/jimmunol.1601279 27815424 PMC5114884

[47] Erlebacher A: Immunology of the maternal-fetal interface. *Annu Rev Immunol.* 2013;31:387–411. 10.1146/annurev-immunol-032712-100003 23298207

[48] Tafuri A Alferink J Möller P: T cell awareness of paternal alloantigens during pregnancy. *Science.* 1995;270(5236):630–3. 10.1126/science.270.5236.630 7570020

[49] Hsu P Nanan RK: Innate and adaptive immune interactions at the fetal-maternal interface in healthy human pregnancy and pre-eclampsia. *Front Immunol.* 2014;5:125. 10.3389/fimmu.2014.00125 24734032 PMC3975095

[50] Tilburgs T Roelen DL van der Mast BJ: Evidence for a selective migration of fetus-specific CD4 ^+^CD25 ^bright^ regulatory T cells from the peripheral blood to the decidua in human pregnancy. *J Immunol.* 2008;180(8):5737–45. 10.4049/jimmunol.180.8.5737 18390759

[51] Mjösberg J Berg G Jenmalm MC: FOXP3 ^+^ regulatory T cells and T helper 1, T helper 2, and T helper 17 cells in human early pregnancy decidua. *Biol Reprod.* 2010;82(4):698–705. 10.1095/biolreprod.109.081208 20018909

[52] Djurisic S Skibsted L Hviid TV: A Phenotypic Analysis of Regulatory T Cells and Uterine NK Cells from First Trimester Pregnancies and Associations with HLA-G. *Am J Reprod Immunol.* 2015;74(5):427–44. 10.1111/aji.12421 26293482

[53] Giugliano S Petroff MG Warren BD: Hepatitis C Virus Sensing by Human Trophoblasts Induces Innate Immune Responses and Recruitment of Maternal NK Cells: Potential Implications for Limiting Vertical Transmission. *J Immunol.* 2015;195(8):3737–47. 10.4049/jimmunol.1500409 26342030 PMC4592818

[54] Quillay H El Costa H Duriez M: NK cells control HIV-1 infection of macrophages through soluble factors and cellular contacts in the human decidua. *Retrovirology.* 2016;13(1):39. 10.1186/s12977-016-0271-z 27267272 PMC4895978

[55] Vermillion MS Lei J Shabi Y: Intrauterine Zika virus infection of pregnant immunocompetent mice models transplacental transmission and adverse perinatal outcomes. *Nat Commun.* 2017;8: 14575. 10.1038/ncomms14575 28220786 PMC5321801

